# Association between the G-protein-coupled estrogen receptor 1 and prostate cancer in the Slovak population

**DOI:** 10.1007/s11033-026-12131-z

**Published:** 2026-06-18

**Authors:** Martina Mečiaková, Ján Kliment, Róbert Dušenka, Daniel Evin, Martina Knoško Brožová, Monika Kmeťová Sivoňová, Dušan Dobrota, Jana Jurečeková

**Affiliations:** 1 Department of Medical Biochemistry, Jessenius Faculty of Medicine in Martin, Comenius University in Bratislava, Malá Hora 4D, Martin, 03601 Slovakia; 2 Department of Urology, Jessenius Faculty of Medicine and University Hospital Martin, Comenius University in Bratislava, Kollárova 2, Martin, 03659 Slovakia; 3 Clinic of Nuclear Medicine, Jessenius Faculty of Medicine and University Hospital Martin, Comenius University in Bratislava, Kollárova 2, Martin, 03659 Slovakia

**Keywords:** Prostate cancer, G-protein coupled estrogen receptor 1, genetic alterations, rs3808350, rs3808351, rs11544331

## Abstract

**Background:**

Prostate cancer ranks as the most diagnosed oncological disease in Slovak men and the third most common cause of their cancer related mortality. G-protein coupled estrogen receptor 1 (GPER1) is estrogen receptor mediating fast non-genomic estrogen signaling and shown potential as therapeutic target for later stages of the disease and in chemoprevention. However, little is currently known about the impact of genetic variability in the GPER1 gene on the prostate cancer risk and disease characteristics.

**Methods and results:**

Genomic DNA samples isolated from peripheral blood of 701 prostate cancer patients and 659 healthy men were used for the single nucleotide polymorphism (SNP) analysis. The presence of GPER1 SNPs (rs3808350, rs3808351, rs11544331) was determined by ARMS-PCR. GPER1 expression was quantified by semi-quantitative RT-PCR using mRNA isolated from prostate cancer tissues and using benign prostate hyperplasia tissues as a control. The GPER1 SNP rs3808350 was associated with a higher risk of developing prostate cancer, especially in patients with a Gleason score ≥ 7 and with nodal and distant metastases. No association was found between the rs3808351 and rs11544331 and prostate cancer. No significant changes in GPER1 expression were found.

**Conclusion:**

The GPER1 rs3808350 was associated with a higher risk of prostate cancer as well as with its characteristics and appears to influence prostate cancer susceptibility and aggressiveness. In the future, these findings could help in personalized treatment and prognosis determination.

## Introduction

Prostate cancer accounts for approximately 15% of all cancers affecting men [1]. According to the Global Cancer Observatory, prostate cancer is the most commonly diagnosed type of cancer in Slovak men in 2022, and the third most common cause of cancer-related death in men in Slovakia [2]. The main risk factors of prostate cancer are genetics, family history, race and especially age [3]. Diagnosis begins with a digital rectal examination and analysis of PSA levels in the blood. Biopsy is required to confirm the presence of the disease [4]. Prostate cancer is hormone-sensitive and depends on the male sex steroid hormones, androgens. This dependency is targeted with androgen deprivation therapy (ADT), which usually utilizes androgen depletion and inhibition of the nuclear androgen receptor (AR), which alters androgen signaling. Although ADT is initially effective, patients inevitably develop resistance and progress to lethal castration-resistant prostate cancer (CRPC) with limited treatment options [5].

Estrogens can also influence prostate cancer pathology, as was firstly demonstrated in the pioneering study by Huggins and Hodges in 1941 [6]. Estrogen therapy was used as a hormonal treatment to slow down the progression of prostate cancer, but was later discontinued due to numerous side effects [7]. Estradiol (E2) is the primary estrogen in men, which serum level range from 10 to 40 pg/mL [8]. Approximately a quarter of E2 is produced by the testes, while the remainder comes from aromatization of testosterone in peripheral tissue, particularly adipose tissue. The prostate stroma also utilizes aromatase to synthesize estrogens [9]. The action of estrogen is mediated by nuclear and membrane receptors. The main nuclear receptors are ERα and ERβ, which act as transcription factors and play different roles in prostate cancer. ERα is tumorigenic and promotes proliferation and inflammation. ERβ acts as a tumor suppressor and its loss is associated with cancer progression [10]. G protein-coupled estrogen receptor 1 (GPER1) mediates rapid non-genomic signaling by activating intracellular signaling pathways [11]. GPER1 is encoded by the GPER1 gene located on chromosome 7p22.3 and consisting of 3 exons. GPER1 protein consists of 375 amino acids and weighs approximately 41 kDa. It has the characteristic heterotrimeric structure consisting of seven transmembrane α-helical regions, four extracellular segments and four cytosolic segments. Receptor activation and ligand binding occur at the extracellular N-terminal end, which is also the site of glycosylation. The intracellular C-terminal end contains a PDZ domain, which is important for cell membrane localization and interaction with plasma membrane proteins. The C-terminal region is also involved in receptor desensitization and internalization depending on phosphorylation by G protein-coupled receptor kinases [7]. GPER1 utilizes multiple G proteins and epidermal growth factor (EGF) receptor transactivation to activate signaling cascades. GPER1 can activate adenylyl cyclase, ERK1/2, PI3K–Akt and other signaling pathways [11]. Under normal conditions, GPER1 is located at the cell membrane, in the endoplasmic reticulum, but also in the nucleus and is widely expressed in various tissues, suggesting the functional versatility of this receptor [12]. GPER1 is expressed in the brain, where regulates serotonin receptor signaling and gonadal function, and is involved in neuroprotection against neurodegenerative diseases [13, 14]. In addition, GPER1 helps maintain cardiac function and reduces inflammation [15]. This receptor has also a role in the regulation of metabolism and has been associated with metabolic diseases such as diabetes or obesity [16]. In addition, GPER1 has been associated with multiple types of cancer [12]. GPER1 is expressed in 50–60% of breast cancer tissues and its expression is increased in triple-negative breast cancer. Its expression was positively correlated with HER2/neu status, tumor size, metastasis, and was also associated with tamoxifen resistance. However, there are also results indicating its tumor-suppressive function [17]. Conflicting results regarding the role of GPER1 in carcinogenesis have also been reported in ovarian and endometrial cancer [12, 18]. GPER1 is expressed in both normal prostate and prostate cancer. In normal prostate, GPER1 was localized predominantly in the cytoplasm, and receptor expression was reported in epithelial cells with the exception of luminal secretory cells and was also observed in stromal cells [19]. In prostate cancer, GPER1 was localized in the cytoplasm, but could also be detected in the nucleus. Furthermore, GPER1 was observed in both tumor epithelium and stromal tissue [19–21]. GPER1 expression was found to be decreased in prostate cancer and seems to be related to Gleason score. GPER1 appears to have tumor suppressor role in prostate cancer [22–24]. Chan and colleagues reported that GPER1, upon activation by the selective agonist G-1, led to cell cycle arrest through activation of the Erk1/2 pathway [23]. Lam and colleagues also reported that use of G-1 resulted in tumor growth inhibition and necrosis [22]. On the other hand, GPER1 expression was higher in metastatic castration-resistant prostate cancer. The GPER1 gene contains an androgen receptor binding site that appears to inhibit GPER1 expression and therefore castration might result in increased GPER1 expression [22]. The function of GPER1 might differ depending on the prostate compartment. GPER1 may mediate a proliferative effect on stromal cells [25–27]. In epithelial cells, GPER1 seemed to impair proliferation [28, 29]. So far, GPER1 has shown potential as a therapeutic target for resistant prostate cancer and as a chemoprevention for this disease [21, 22].

Single-nucleotide polymorphisms (SNPs) can alter gene expression, protein stability or function. Although multiple SNPs were identified in the GPER1 gene, only three are considered biologically relevant: rs3808350, rs3808351, and rs11544331. Both rs3808350 and rs3808351 are located in the promoter region of the GPER1 gene and could therefore alter the affinity for transcription factors and thus the expression of the GPER1 gene. Studies of the exact functional impact of these polymorphisms are currently unavailable. The rs11544331 is localized in exon 3 and results in an amino acid change from proline to leucine [30, 31]. It is thought that rs11544331 affects the localization of GPER1. The replacement of proline with leucine results in the loss of glycosylation site and consequently controls the nuclear localization of GPER1 [32]. GPER1 SNPs have been studied in various diseases, including cancer; however, to date, no studies have examined the association between GPER1 SNPs and prostate cancer [30, 31]. In our study, we aim to evaluate the association between GPER1 SNPs and the risk of prostate cancer development and progression. The present study will also determine the possible impact of these SNPs on the histopathological characteristics of prostate cancer that are routinely used to describe this disease.

## Materials and methods

### Study population

The present work is a case-control study approved by the Ethics Committee of Comenius University in Bratislava, Jessenius Faculty of Medicine in Martin, and conducted in accordance with the Declaration of Helsinki. All participants were enrolled at the Department of Urology, University Hospital Martin in Slovakia between 2005 and 2023, and provided written informed consent to participate in the study. The criteria for inclusion in the patient group were as follows: (1) age ≥ 50 years; (2) Caucasians; (3) histologically confirmed prostate cancer. Patients with previous or current evidence of other oncological disease or other serious pathologies were excluded. The criteria for inclusion in the control group were as follows: (1) age ≥ 50 years; (2) Caucasians; (3) no current or previous diagnoses of any oncological disease; (4) negative serum PSA level according to age-specific reference values.

The calculated required sample size to achieve 80% power with the effect size of at least 0.1 at the 0.05 significance level was 964 samples in total. To account for the nature of the data generation process and potential drop-out, the sample size was increased by 40%. A total number of 1360 men were included in study, representing 92% statistical power according to the same criteria. Participants were divided into the patient group consisting of 701 men with prostate cancer and the control group consisting of 659 healthy men. Characteristics of the study population are summarized in Table 1. Tissue samples were obtained from 45 patients with prostate cancer and 40 men with benign prostatic hyperplasia (BPH).

**Table 1:** Characterization of control group and patient group

Characteristics	Control (*n* = 659)	Prostate cancer (*n* = 701)
Age (years)		
Mean ± SD	64.80 ± 10.51	67.92 ± 8.46
PSA, ng/ml		
Median (25–75th percentile)	2.16 (0.82–4.85)	12.00 (6.49-38.375)
Gleason score		
< 7	NA	127
≥ 7	NA	481
Mean ± SD	NA	7.40 ± 1.22
Missing	NA	93
Pathological stage		
pT1/pT2	NA	136
pT3/pT4	NA	258
Missing	NA	307
Nodal involvement		
N0	NA	305
N1	NA	50
Missing	NA	346
Distant metastasis		
M0	NA	136
M1	NA	123
Missing	NA	442

SD – standard deviation, NA – not applicable

### DNA isolation

Genomic DNA used for genotyping was isolated from blood samples obtained from participants. For genomic DNA isolation, we used the Wizard^®^ Genomic DNA Purification kit (Promega Corporation) according to the manufacturer’s protocol, and genomic DNA was further stored at − 20 °C until further analysis.

### Total RNA isolation

For total RNA isolation, we used tissue samples collected during routine surgery, which were stored in RNA stabilization solution at − 80 °C. Isolation of total RNA was performed using an AllPrep DNA/RNA/miRNA Universal kit (Qiagen GmbH) according to the manufacturer’s protocol.

### Genotyping

All *GPER1* polymorphisms were analyzed using tetra-primer amplification refractory mutation system PCR (ARMS-PCR). The *GPER1* polymorphism rs3808350 was determined using the following primers: P1–5’-CTA TTT TTA AGT GAC ATG TCG CA-3‘, P2–5’-TAA AAA TTC AAA CCT TGA AAT ATC C3-’, P3–5’-CAG TAC AAG TTA CTT ACC CGC C-3’, P4–5’-ATA TGT ACC TTT TTG TAT TTG GAT GAT A-3’. The PCR master mix contained 100 ng genomic DNA, 2,4 µl 5x HOT FIREPol^®^ Blend Master Mix RTL (Solis BioDyne OÜ), 0.5 µl of each primer, and 6.6 µl nuclease-free water. The ARMS-PCR was performed under the following conditions: initial denaturation at 95 °C for 15 min followed by 37 cycles of denaturation at 95 °C for 20 s, primer annealing at 58 °C for 45 s, and extension at 72 °C for 1 min, with a final extension at 72 °C for 5 min. The allele-specific products of the following size: 405 bp for the outer primer product, 294 bp for the G allele, and 205 bp for the A allele were separated with 3% agarose gel electrophoresis. The *GPER1* polymorphism rs3808351 was determined using the following primers: P1–5’-CGC TTG GGG GGC CTC GCT ATG-3’, P2–5’-CGA TGG CCG CCC CAT GAG TGT-3’, P3–5’-CTC ATA CTC AGC GGA CAA AGG ATC ACT CAG C-3’, P4–5’-CTG CTC CTG GTT GCG GAT TTC ACA GTC T-3’. The PCR master mix contained 100 ng genomic DNA, 2,4 µl 5x HOT FIREPol^®^ Blend Master Mix RTL (Solis BioDyne OÜ), 0.5 µl of P1 and P2 primers, 0.3 µl of P3 and P4 primers, and 7 µl nuclease-free water. The ARMS-PCR was performed under the following conditions: initial denaturation at 95 °C for 15 min followed by 35 cycles of denaturation at 95 °C for 20 s, primer annealing at 67.3 °C for 45 s, and extension at 72 °C for 1 min, with a final extension at 72 °C for 5 min. The allele-specific products of the following size: 385 bp for the outer primer product, 231 bp for the A allele, and 196 bp for the G allele were separated with 4% agarose gel electrophoresis. The *GPER1* SNP rs11544331 was determined using the following primers: P1–5’-GGG CGT GGG CCT GGA GAT GTA ACC-3’, P2–5’-CGC AGG CTG CGC GGT GCA TA-3’, P3–5’-AAC AAA CCC AAC CCA AAC CAC CAC AGG T-3’, P4–5’-AGC CGA TGG GGA AGA GGA AGA TGG TGT A-3’. The PCR master mix contained 100 ng genomic DNA, 2,4 µl 5x HOT FIREPol^®^ Blend Master Mix RTL (Solis BioDyne OÜ), 0.5 µl of P1 and P2 primers, 0.3 µl of P3 and P4 primers, and 7 µl nuclease-free water. The ARMS-PCR was performed under the following conditions: initial denaturation at 95 °C for 15 min, followed by 35 cycles of denaturation at 95 °C for 20 s, primer annealing at 68 °C for 45 s, and extension at 72 °C for 1 min, with a final extension at 72 °C for 5 min. The allele-specific products of the following size: 391 bp for the outer primer product, 237 bp for the T allele, and 198 bp for the C allele were separated with 4% agarose gel electrophoresis.

### Gene expression analysis

For each sample, an equal quantity of RNA (1 µg) was used for reverse transcription into cDNA with a RT2 First Strand kit, following the standard protocol (Qiagen GmbH). Reverse transcription-quantitative PCR analysis (RT-qPCR) of the *GPER1* expression was performed with the RT^2^ qPCR primer assay (Qiagen GmbH) using *GAPDH* as a housekeeping gene.

### Statistical analysis

We used the chi-square test to evaluate the Hardy-Weinberg equilibrium. Odds ratios (OR), corresponding 95% confidence intervals (CI) and p-values (p) were calculated with logistic regression model. All the results were adjusted using age. The mRNA expression of the *GPER1* gene was analyzed using a two-sample, two-sided t-test on log2-transformed fold change (FC) data. We considered all p-values below 0.05 as statistically significant. Statistical analysis was performed using the StatsDirect statistical package version 2.7.0.2.

## Results

All three analyzed *GPER1* polymorphisms were in Hardy-Weinberg equilibrium (*p* > 0.05). We evaluated the effect of *GPER1* polymorphisms on prostate cancer susceptibility and disease features in codominant, dominant, recessive, and allelic models. We detected a statistically significant association between prostate cancer susceptibility and the *GPER1* rs3808350 in the dominant model (OR = 1.42, CI = 1.13–1.78, *p* = 0.002) and the variant G allele of rs3808350 in the allelic model (OR = 1.27, CI = 1.09–1.49, *p* = 0.003). We also found a significant association between prostate cancer susceptibility and the rs3808350 AG (OR = 1.38, CI = 1.09–1.75, *p* = 0.008) and GG genotypes in codominant model (OR = 1.56, CI = 1.12–2.18, *p* = 0.009). We did not observe any association between prostate cancer susceptibility and *GPER1* rs3808351 or rs11544331 polymorphisms in any analyzed model (Table 2).

After stratification of patients according to Gleason score, that assesses how much the prostate cancer tissue has transformed from healthy form to malignant [33], we found that *GPER1* polymorphism rs3808350 was associated with the development of prostate cancer with Gleason score < 7 (OR = 1.55, CI = 1.03–2.34, *p* = 0.04) as well as with the development of prostate cancer with Gleason score ≥ 7 (OR = 1.40, CI = 1.09–1.80, *p* = 0.008) in the dominant model. In the codominant model, the rs3808350 AG (OR = 1.35, CI = 1.04–1.76, *p* = 0.03) and GG genotypes (OR = 1.59, CI = 1.10–2.29, *p* = 0.01) were associated with the development of prostate cancer with Gleason score ≥ 7. In the allelic model, the G allele of rs3808350 was associated with the development of prostate cancer with Gleason score ≥ 7 (OR = 1.27, CI = 1.07–1.52, *p* = 0.006) (Table 3). We did not find any association between the other two *GPER1* polymorphisms and Gleason scores in any of the studied model (data not shown).

**Table 2:** Distribution of GPER1 genotypes and alleles and their association with the risk of prostate cancer

Genotype	Control, *n*	Prostate cancer, *n*		OR (95% CI)	*p*-value
**GPER rs3808350**					
*Codominant model*					
AA	264		224	1.00 (Ref.)	
AG	303		359	**1.38 (1.09–1.75)**	**0.008** ^**a**^
GG	91		118	**1.56 (1.12–2.18)**	**0.009** ^**a**^
*Dominant model*					
AA	264		224	1.00 (Ref.)	
AG + GG	394		477	**1.42 (1.13–1.78)**	**0.002** ^**a**^
*Recessive model*					
AA + AG	567		583	1.00 (Ref.)	
GG	91		118	1.29 (0.95–1.74)	0.10
*Allelic model*					
A	831		807	1.00 (Ref.)	
G	485		595	**1.27 (1.09–1.49)**	**0.003** ^**a**^
**GPER rs3808351**					
*Codominant model*					
GG	308		317	1.00 (Ref.)	
AG	283		291	1.00 (0.79–1.25)	0.97
AA	61		76	1.03 (0.83–1.29)	0.75
*Dominant model*					
GG	308		317	1.00 (Ref.)	
AG + AA	344		367	1.04 (0.83–1.29)	0.75
*Recessive model*					
GG + AG	591		608	1.00 (Ref.)	
AA	61		76	1.24 (0.86–1.77)	0.25
Allelic model					
G	899		925	1.00 (Ref.)	
A	405		443	1.07 (0.91–1.26)	0.44
**GPER rs11544331**					
Codominant model					
CC	348		394	1.00 (Ref.)	
CT	271		273	0.89 (0.71–1.11)	0.29
TT	40		34	0.87 (0.70–1.08)	0.20
Dominant model					
CC	348		394	1.00 (Ref.)	
CT + TT	311		307	0.87 (0.70–1.08)	0.20
Recessive model					
CC + CT	619		667	1.00 (Ref.)	
TT	40		34	0.78 (0.49–1.26)	0.32
Allelic model					
C	967		1061	1.00 (Ref.)	
T	351		341	0.88 (0.73–1.05)	0.15

After stratification of patients according to PSA, which high levels are considered to be a sign of prostate cancer [34], we observed that the rs3808350 was associated with the higher risk of development of prostate cancer in patients with PSA levels < 10 ng/ml in all tested models (Table 4). The rs3808350 was also associated with the development of prostate cancer in patients with PSA levels **≥** 10 ng/ml in dominant model (OR = 1.37, CI = 1.04–1.81, *p* = 0.02) (Table 4). We did not identify any association between the other two *GPER1* polymorphisms and PSA levels (data not shown).

**Table 3:** Association of GPER1 rs3808350 genotypes and alleles with Gleason score

GPER rs3808350	Control	Prostate cancer Gleason score < 7			Prostate cancer Gleason score ≥ 7		
	*n*	*n*	OR (95% CI)	*p*-value	*n*	OR (95% CI)	*p*-value
Codominant model							
AA	264	38	1.00 (Ref.)		155	1.00 (Ref.)	
AG	303	69	1.55 (1.01–2.39)	0.05	244	**1.35 (1.04–1.76)**	**0.03** ^**a**^
GG	91	20	1.54 (0.85–2.79)	0.16	82	**1.59 (1.10–2.29)**	**0.01** ^**a**^
Dominant model							
AA	264	38	1.00 (Ref.)		155	1.00 (Ref.)	
AG + GG	394	89	**1.55 (1.03–2.34)**	**0.04** ^**a**^	326	**1.40 (1.09–1.80)**	**0.008** ^**a**^
Recessive model							
AA + AG	567	107	1.00 (Ref.)		399	1.00 (Ref.)	
GG	91	20	1.18 (0.70-2.00)	0.54	82	1.33 (0.96–1.84)	0.09
Allelic model							
A	831	145	1.00 (Ref.)		554	1.00 (Ref.)	
G	485	109	1.29 (0.98–1.70)	0.07	408	**1.27 (1.07–1.52)**	**0.006** ^**a**^

^a^ Statistically significant results (p value < 0.05)

**Table 4:** Association of GPER1 rs3808350 genotypes and alleles with PSA levels

GPER rs3808350	Control	Prostate cancer PSA < 10 ng/ml			Prostate cancer PSA ≥ 10 ng/ml		
	*n*	*n*	OR (95% CI)	*p*-value	*n*	OR (95% CI)	*p*-value
Codominant model							
AA	264	85	1.00 (Ref.)		119	1.00 (Ref.)	
AG	303	127	1.29 (0.93–1.78)	0.12	192	1.39 (1.04–1.85)	**0.03** ^**a**^
GG	91	50	**1.71 (1.12–2.61)**	**0.01** ^**a**^	53	1.34 (0.88–2.03)	0.17
Dominant model							
AA	264	85	1.00 (Ref.)		119	1.00 (Ref.)	
AG + GG	394	177	**1.38 (1.02–1.87)**	**0.03** ^**a**^	245	**1.37 (1.04–1.81)**	**0.02** ^**a**^
Recessive model							
AA + AG	567	212	1.00 (Ref.)		311	1.00 (Ref.)	
GG	91	50	**1.48 (1.01–2.16)**	**0.047** ^**a**^	53	1.09 (0.75–1.59)	0.64
Allelic model							
A	831	297	1.00 (Ref.)		430	1.00 (Ref.)	
G	485	227	**1.30 (1.06–1.60)**	**0.01** ^**a**^	298	1.20 (0.99–1.46)	0.06

^a^ Statistically significant results (p value < 0.05)

In the dominant model, rs3808350 was associated with the development of prostate cancer with pT1/pT2 stage (OR = 1.54, CI = 1.03–2.29, *p* = 0.03) as well as with prostate cancer with pT3/pT4 stage (OR = 1.55, CI = 1.13–2.13, *p* = 0.006). In the codominant model, the AG genotype of rs3808350 was associated with the development of prostate cancer with pT1/pT2 stage (OR = 1.55, CI = 1.02–2.36, *p* = 0.04) as well as with prostate cancer with pT3/pT4 stage (OR = 1.56, CI = 1.12–2.18, *p* = 0.008). In the allelic model, the G allele of rs3808350 was associated with the development of prostate cancer with pT3/pT4 stage (OR = 1.29, CI = 1.04–1.61, *p* = 0.02) (Table 5). *GPER1* rs3808351 and rs11544331 polymorphisms were not associated the development of prostate cancer after stratification of patients based on pathological T stage (data not shown).

Next, we analyzed the association of *GPER1* polymorphisms with prostate cancer in patients stratified according to nodal involvement (N0 – without nodal metastasis, N1 – with nodal metastasis). We found that rs3808350 is associated with the higher risk of development of N0 prostate cancer (OR = 1.58, CI = 1.17–2.12, *p* = 0.002) and with the development of N1 prostate cancer (OR = 2.62, CI = 1.29–5.33, *p* = 0.004) in the dominant model. In the codominant model, the rs3808350 AG genotype was associated with a higher risk of development of N0 prostate cancer (OR = 1.63, CI = 1.20–2.22, *p* = 0.002) and with N1 prostate cancer (OR = 2.62, CI = 1.26–5.47, *p* = 0.01), but the rs3808350 GG genotype was associated just with the higher risk of development of N1 prostate cancer (OR = 2.60, CI = 1.02–6.60, *p* = 0.04). In the allelic model, only the rs3808350G allele was associated with the development of N0 prostate cancer (OR = 1.27, CI = 1.03–1.56, *p* = 0.02) and with the development of N1 prostate cancer (OR = 1.65, CI = 1.09–2.50, *p* = 0.02) (Table 6). There was no statistically significant association between *GPER1* rs3808351 or rs11544331 polymorphisms and nodal involvement (data not shown).

**Table 5:** Association of GPER1 rs3808350 genotypes and alleles with pathological stage

GPER rs3808350	Control	Prostate cancer pT1/pT2			Prostate cancer pT3/pT4		
	*n*	*n*	OR (95% CI)	*p*-value	*n*	OR (95% CI)	*p*-value
Codominant model							
AA	264	41	1.00 (Ref.)		76	1.00 (Ref.)	
AG	303	74	**1.55 (1.02–2.36)**	**0.04** ^**a**^	142	**1.56 (1.12–2.18)**	**0.008** ^**a**^
GG	91	21	1.49 (0.84–2.66)	0.18	40	1.53 (0.96–2.44)	0.07
Dominant model							
AA	264	41	1.00 (Ref.)		76	1.00 (Ref.)	
AG + GG	394	95	**1.54 (1.03–2.29)**	**0.03** ^**a**^	182	**1.55 (1.13–2.13)**	**0.006** ^**a**^
Recessive model							
AA + AG	567	115	1.00 (Ref.)		218	1.00 (Ref.)	
GG	91	21	1.15 (0.68–1.92)	0.61	40	1.17 (0.77–1.77)	0.46
Allelic model							
A	831	156	1.00 (Ref.)		294	1.00 (Ref.)	
G	485	116	1.28 (0.98–1.67)	0.08	222	**1.29 (1.04–1.61)**	**0.02** ^**a**^

^a^ Statistically significant results (p value < 0.05)

**Table 6:** Association of GPER1 rs3808350 genotypes and alleles with nodal involvement

GPER rs3808350	Control	Prostate cancer N0			Prostate cancer N1		
	*n*	*n*	OR (95% CI)	*p*-value	*n*	OR (95% CI)	*p*-value
Codominant model							
AA	264	90	1.00 (Ref.)		10	1.00 (Ref.)	
AG	303	172	**1.63 (1.20–2.22)**	**0.002** ^**a**^	31	**2.62 (1.26–5.47)**	**0.01** ^**a**^
GG	91	43	1.41 (0.90–2.20)	0.13	9	**2.60 (1.02–6.60)**	**0.04** ^**a**^
Dominant model							
AA	264	90	1.00 (Ref.)		10	1.00 (Ref.)	
AG + GG	394	215	**1.58 (1.17–2.12)**	**0.002** ^**a**^	40	**2.62 (1.29–5.33)**	**0.004** ^**a**^
Recessive model							
AA + AG	567	262	1.00 (Ref.)		41	1.00 (Ref.)	
GG	91	43	1.05 (0.70–1.56)	0.83	9	1.38 (0.65–2.95)	0.41
Allelic model							
A	831	352	1.00 (Ref.)		51	1.00 (Ref.)	
G	485	258	**1.27 (1.03–1.56)**	**0.02** ^**a**^	49	**1.65 (1.09–2.50)**	**0.02** ^**a**^

^a^ Statistically significant results (p value < 0.05)

Finally, we analyzed the association between *GPER1* polymorphisms and the presence of distant metastases (M0 – without distant metastases, M1 – with distant metastases). We found out that the rs3808350 polymorphism was associated with higher risk of development of M1 prostate cancer in dominant model (OR = 1.64, CI = 1.06–2.54, *p* = 0.02), while in codominant model only the rs3808350 AG genotype was associated with higher risk of development of M1 prostate cancer (OR = 1.73, CI = 1.11–2.72, *p* = 0.02) (Table 7). We did not observe any association between the rs3808351 and rs11544331 polymorphisms and distant metastases in any evaluated model (data not shown).

**Table 7:** Association of GPER1 rs3808350 genotypes and alleles with presence of distant metastases

GPER rs3808350	Control	Prostate cancer M0			Prostate cancer M1		
	*n*	*n*	OR (95% CI)	*p*-value	*n*	OR (95% CI)	*p*-value
Codominant model							
AA	264	43	1.00 (Ref.)		34	1.00 (Ref.)	
AG	303	72	1.38 (0.90–2.13)	0.14	73	**1.73 (1.11–2.72)**	**0.02** ^**a**^
GG	91	21	1.50 (0.82–2.75)	0.19	16	1.34 (0.69–2.60)	0.38
Dominant model							
AA	264	43	1.00 (Ref.)		34	1.00 (Ref.)	
AG + GG	394	93	1.40 (0.93–2.12)	0.10	89	**1.64 (1.06–2.54)**	**0.02** ^**a**^
Recessive model							
AA + AG	567	115	1.00 (Ref.)		107	1.00 (Ref.)	
GG	91	21	1.23 (0.72–2.11)	0.46	16	0.96 (0.53–1.72)	0.88
Allelic model							
A	831	158	1.00 (Ref.)		141	1.00 (Ref.)	
G	485	114	1.25 (0.94–1.66)	0.12	105	1.26 (0.94–1.69)	0.13

^a^ Statistically significant results (p value < 0.05)

**Fig. 1:**
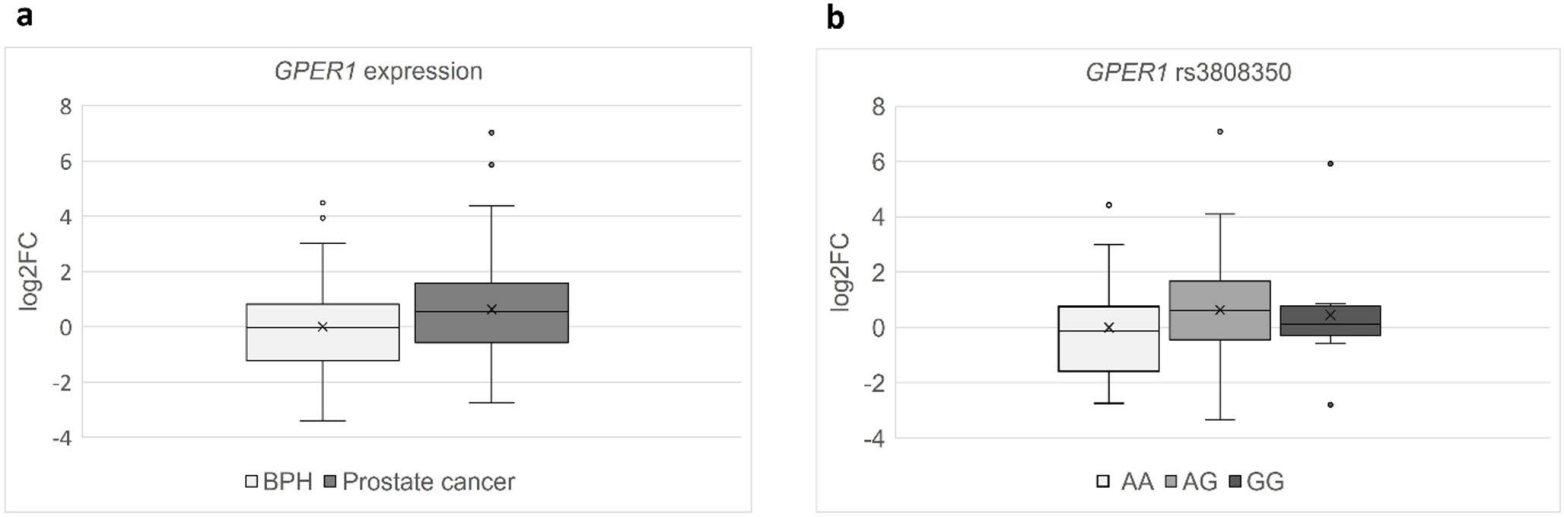
Log_2_ fold change (FC) in the relative mRNA expression of *GPER1* in prostate cancer and benign prostate hyperplasia (BPH) tissues** (a) **and in patients with different rs3808350 genotypes **(b)**. *Boxplots depict the first quartile and third quartile values as lower and upper edges*,* median as horizontal line inside the box. Whiskers depict the minimal and maximal values*,* unless there are outliers in the data*

The *GPER1* mRNA expression was detected in all analyzed prostate cancer tissues (mean Ct value for *GPER1* was 31.68 and mean Ct value for *GAPDH* was 23.08) and BPH tissues (mean Ct value for *GPER1* was 32.54 and mean Ct value for *GAPDH* was 23.30). We found that the relative *GPER1* mRNA expression levels were 1.54-fold higher in prostate cancer tissues compared to BPH tissues (Fig. 1). We further analyzed the relative *GPER1* mRNA expression levels based on the presence of *GPER1* polymorphisms. Patients with the variant rs3808350 GG genotype had 1.71-fold higher GPER1 mRNA expression compared to patients with the AA genotype (Fig. 1). We also observed that patients with the variant rs3808351 AA genotype had 1.29-fold higher expression of GPER1 mRNA than patients with GG genotype. Patients with the rs11544331 TT genotype had 1.47-fold lower relative expression of GPER1 mRNA than patients with CC genotype (data not shown). None of these results were statistically significant.

## Discussion

Prostate cancer is influenced by hormonal regulation. While androgens are the main factors leading to the development of this disease, estrogens also affect the prostate gland, its growth and differentiation and development of prostate cancer. The effect of estrogens is mediated by estrogen receptors. Nuclear ERs are transcriptional factors leading to genomic signaling, while GPER1 mediates non-genomic estrogen signaling through the activation of signaling cascades [35]. To the best of our knowledge, this is the first study of a possible association between *GPER1* rs3808350, rs3808351, and rs11544331 polymorphisms and prostate cancer risk. However, these polymorphisms were previously investigated in several diseases, ranging from benign (gynecomastia, uterine leiomyoma) to malignant (breast cancer, seminoma) [30, 36–39].

In the case of rs3808350, we observed that the both AG and GG genotypes were significantly associated with the increased risk of development of prostate cancer as well as with prostate cancer clinical features such as Gleason score, PSA levels, pathological tumor stage, nodal status, and metastatic status. The role of the rs3808350 polymorphism has not been previously investigated in prostate cancer, but has been examined in other cancers. The GG genotype was found to be associated with a higher risk of the development of uterine leiomyoma [37, 39]. Conversely, in testicular seminoma, the GG genotype showed a protective effect against this cancer [36]. No association was found between overall breast cancer risk and rs38080350, but homozygous GG genotype of this polymorphism was associated with progesterone receptor status with a potential protective effect regarding the development of progesterone-negative breast cancer [30]. Meta-analysis from 2021 reported no association between GPER1 rs3808350, rs3808351 and rs11544331 SNPs and overall cancer susceptibility, but after population stratification, they observed a statistically significant association between rs3808350 and cancer susceptibility in Asian population, while rs3808351 was associated with a lower risk of cancer in the same population. No association was found between rs11544331 and cancer predisposition after the population stratification. Based on this, it could be argued that the impact of these SNPs may vary between different populations [31].

Although the previous meta-analysis suggested that rs3808351 may influence cancer predisposition, with the A allele being protective, in our study we did not observe any association between rs3808351 and prostate cancer or selected clinical features of prostate cancer. On the other hand, the G allele as well as the GG genotype, were reported to be associated with a higher risk of uterine leiomyoma [31, 37, 39]. The AA genotype was also more common in patients with seminoma than in healthy men, and the GG genotype has been shown to be protective against this disease [36]. Rs3808351 did not directly influence the risk of breast cancer, but was associated with tumor size and histological grading. The AG and GG genotypes were found to be significantly less common in patients with large tumors. The A allele was significantly less prevalent in patients with G3 grade compared to patients with G2 breast cancer or healthy women [30].

We also did not find any association between rs11544331 and the risk of prostate cancer. This is in line with previous reports in which rs11544331 was not associated with general cancer predisposition, testicular cancer, or breast cancer [30, 31, 36]. Overexpression of this GPER1 polymorphism stimulates the migration of cancer-associated fibroblasts and might be a mechanism contributing to the development of metastasis [32]. Despite this fact, the variant T allele was less common in breast cancer patients with nodal metastasis [30, 32].

GPER1 expression can be observed in virtually all organs and its deregulation contributes to the development of several diseases, from benign to malignant [11]. In the prostate, GPER1 is expressed in epithelial and stromal cells and can be found in both cytoplasmic and nuclear compartments [19, 20]. GPER1 expression changes during disease progression and generally decreases with increasing Gleason score. However, GPER1 expression can increase again at the stage of fatal castration resistance [19, 22]. We observe a trend of increased relative *GPER1* mRNA expression in prostate cancer tissues. This could be due to our patient cohort, which consisted of patients with prostate cancer at different stages. Both rs3808350 and rs3808351 are localized in the promoter region of the *GPER1* gene and could therefore affect *GPER1* transcription. To date, most studies have focused only on the clinical significance of the *GPER1* SNPs, and the exact impact of rs3808350 and rs3808351 on transcription is currently unknown and only hypothetical. The only functional study involving GPER1 SNPs reported altered receptor functionality affected by rs11544331 [31, 32].

## Conclusions

In summary, we found that rs3808350 was positively associated with the risk prostate cancer in the Slovak population, while there was no association between prostate cancer risk and rs3808351 or rs11544331. The rs3808350 promoter polymorphism was also associated with multiple clinical features of prostate cancer, such as Gleason score, PSA levels, pathological T stage, nodal involvement, and distant metastases. As this is the first study evaluating the possible association of these polymorphisms with the risk of prostate cancer development and progression, further studies involving larger and multiethnic populations will be needed to verify the clinical potential of our findings, as well as to investigate their precise functional impact on GPER1 expression.

Authors declare that they have no conflict of interests. All authors contributed to the development and conception of the study. The study approved was by the Ethics Committee of Comenius University in Bratislava, Jessenius Faculty of Medicine in Martin (EK 17/2025), and conducted in accordance with The Declaration of Helsinki. All participants provided written informed consent to participate in the study.

## Data Availability

No datasets were generated or analysed during the current study.

## Abbreviations

ADT: androgen deprivation therapy
Akt: protein kinase B
AR: androgen receptor
BPH: benign prostatic hyperplasia
CI: confidence interval
CRPC: castration-resistant prostate cancer
E2: estradiol
EGF: epidermal growth factor
ERK1/2: extracellular signal-regulated kinase 1/2
ERα: estrogen receptor alpha
ERβ: estrogen receptor b
GPER1: G-protein coupled estrogen receptor 1
M0: prostate cancer without distant metastasis
M1: prostate cancer with distant metastasis
N0: prostate cancer without nodal metastasis
N1: prostate cancer with nodal metastasis
OR: odds ratio
P: P value
PI3K: phoshoinositide 3-kinase
PSA: prostate-specific antigen
SNP: single nucleotide polymorphism
